# Standalone interspinous process devices for lumbar spinal stenosis: a critical literature review

**DOI:** 10.1186/s12893-026-03722-5

**Published:** 2026-04-09

**Authors:** Jimmy Wen, Shannon Dwyer, Megan Kou, Yu-Tung Chen, Allyson Kouche, Sugamjot Badhan, Arsh Alam, Naomi Pai, Megan Diana Hsu, Jose Silva, Foad Elahi

**Affiliations:** 1 California Northstate University College of Medicine, 9700 W Taron Dr, Elk Grove, CA 95757 USA; 2 University of California, Davis, 1 Shields Ave, Davis, CA 95616 USA; 3 University of California, Berkeley, 110 Sproul Hall, Berkeley, CA 94720 USA; 4 Sutter Roseville, Department of Emergency Medicine, 1 Medical Plaza Dr 1st Floor, Roseville, CA 95661 USA; 5 California Center of Pain Medicine and Rehabilitation, 4944 Sunrise Blvd, Fair Oaks, CA 95628 USA

**Keywords:** Interspinous spacers, Fusion, Lumbar spinal stenosis, Funding, Central pain

## Abstract

**Background:**

Interspinous process devices (IPDs) have been widely promoted as a minimally invasive alternative to traditional decompressive surgery for lumbar spinal stenosis (LSS). However, their clinical durability, safety, and comparative effectiveness have remained controversial. This review aims to review and synthesize the biomechanical rationale, clinical outcomes, safety profile, and limitations of IPDs for the treatment of LSS.

**Methods:**

A literature review was conducted using PubMed for clinical studies published between 2000 and 2025 on IPDs. These studies were further categorized by device design, indication, and surgical approach: non-fusion/motion-preserving, fusion/stand-alone, and hybrid/dynamic stabilization.

**Results:**

Biomechanical studies demonstrate that IPDs can consistently increase canal and foraminal dimensions, selectively restrict lumbar extension, and reduce extension-related intradiscal and facet joint pain while preserving motion in other planes. Clinically, short-term improvement was shown particularly in patients with posture-dependent moderate LSS. However, the durability of benefit varies across devices, and several comparative trials and registry-based analyses display higher reoperation and revision rates compared to conventional decompressive surgery. The most commonly reported complications include device migration/malposition and spinous process fractures, leading to revision procedures. Another prevalent issue is the amount of industry funding among IPD studies, which can also influence reported outcomes.

**Conclusion:**

IPDs may provide short-term symptomatic relief for selective patients with LSS, but tend to be associated with variable durability and risks of reintervention. However, long-term benefits in pain, function, or quality of life are currently unclear. Structural correction or indirect decompression alone does not reliably lead to symptomatic relief, especially in patients with chronic radiculopathy or central sensitization. Future studies should consist of large randomized controlled trials that are independently funded to determine comparative effectiveness and optimal patient selection.

## Introduction

Lumbar spinal stenosis (LSS) prevalence is continuously on the rise, with an estimated 11% in the general population and 25% to 39% in clinical settings [1, 2]. This condition is most common in older patients and is a significant cause of disability and indication for spinal surgery in patients older than 65 years [2]. LSS can be acquired or congenital, with the most common cause being degenerative spondylosis due to age, chronic wear and tear, and trauma. Other common causes include intervertebral disc degeneration, degenerative spondylolisthesis, space-occupying lesions, post-surgical sequelae, rheumatologic and autoimmune conditions [2, 3]. Despite the high prevalence of this condition, there is no universally accepted definition or diagnostic criteria [2]. However, one common definition by Henk Verbiest in 1954 is that relative and absolute stenosis is defined as a lumbar canal anteroposterior (AP) diameter of less than 12 mm and 10 mm, respectively [4]. On the other hand, modern imaging criteria typically use computed tomography (CT) cross-sectional area of the spinal sacs less than 75 mm^2^ and 100 mm^2^ to indicate absolute and relative LSS, respectively [2]. Another method is utilizing MRI measurements of the intraspinal canal area of less than 76 mm^2^ and 100 m^2^ are used to indicate severe and moderate LSS, respectively. MRI-grading systems such as the Schizas, Chen Jia, Braz, and Lee grading classifications provide a more comprehensive picture of canal compromise compared to AP diameter alone [2].

Medical management is typically used for short-term pain relief, such as nonsteroidal anti-inflammatory drugs (NSAIDS), opioids, muscle relaxants, gabapentin, and hemp-derived cannabidiol [5]. Physical therapy has low evidence to improve pain scores and function, but it’s important to note that there are no standardized physical therapy regimens for LSS [2]. Bilateral facet injections with steroid or botulinum toxin A are more effective than transforaminal epidural steroid injections, as the latter cannot travel into the spinal canal. Medial branch blocks, along with radiofrequency ablation, have been shown to benefit close to 70% of patients with mild to moderate stenosis, given LSS secondary to spondylosis and facet arthropathy [2].

For patients who fail the above conservative treatments, surgical options are beneficial in up to 80% of patients with severe LSS [2]. The most frequently performed surgery is a pedicle-to-pedicle laminectomy, but the gold standard treatment is the open lumbar decompressive laminectomy. These procedures are indicated for refractory LSS pain after failing conservative treatments for 3 to 6 months. The minimally invasive spine treatment (MIST) guidelines recommend open decompression with or without fusion, with fusion being indicated when further stability is required in patients with concurrent segmental disability [4]. However, no clinical differences have been observed for decompression with and without fusion [6]. There have been improvements in subjective scores, such as the Oswestry Disability Index (ODI), with a 2.55-fold improvement compared to decompression alone [7]. It’s important to note that fusion is more costly, associated with greater blood loss, and longer hospital stays. Additionally, even though there is greater symptomatic improvement compared to patients treated nonsurgically, the benefits tend to diminish over time [8].

Spinal procedures have progressively transitioned toward less invasive methodologies, aiming to reduce the risk of complications while maintaining comparable outcomes to those of open surgical approaches [9]. Finally, interspinous process devices (IPDs) have been used to reduce lumbar extension and are cost-effective, and Food and Drug Administration (FDA) approved for LSS (L1 to L5) involving one or two segments. IPDs are comparable to decompression for pain relief secondary to intermittent neurogenic claudication (NC), benefitting almost 50% of patients [2]. IPDs have been used alone or in conjunction with decompressive surgeries.

IPDs were introduced as an alternative to lumbar fusion and decompression, but do not alter the bony anatomy of the spinal column. IPDs consist of spacers, stabilizers, and standalone fusion devices, and theoretically function by opening the neuroforamina through distracting the spinous processes (“indirect decompression”). This leads to unloading of the facets, decreased intradiscal pressure, and spinal extension to relieve NC symptoms [1]. The first device was approved in 2005 by the FDA, and several similar devices have been introduced since then [4].

However, the scientific evidence remains controversial for its efficacy, limited duration of benefit, high reoperation rates, and cost-effectiveness [10]. An indirect decompression, achieved by increasing the neuroforaminal area mechanically, does not guarantee radicular pain relief, as the pain generators are frequently multifactorial, involving neuropathic and central sensitization mechanisms, rather than mechanical compression alone. Another issue is that the early clinic studies had small sample sizes, low trial quality, missing data for important outcomes, and other high risks of bias, such as unbalanced demographic data or inappropriate funding, with some devices being withdrawn from the market [1, 10].

This narrative review aims to evaluate the literature on standalone IPDs to focus on their biomechanics, clinical outcomes, safety profile, and the differences between mechanical correction and chronic pain pathophysiology.

## Literature search methods

A focused literature search was performed in the PubMed database for all IPD articles published from January 1, 2000, through December 1, 2025. This timeframe was selected as the first successful devices introduced commercially were in the early 2000s and continued to be developed and evaluated in clinical studies throughout this time. The search strategy encompassed the following keywords alone or in combination with MeSH terms (OR, AND): interspinous process device, interspinous fusion, interspinous spacer, interspinous implant, fixation, spacer, implant, X-Stop™, Superion™, Aperius™, Helifix™, Coflex™, Wallis™, DIAM™, Minuteman™, and Spire™.

Studies were included if they met the criteria: (1) published between 2000 and 2025, (2) published in English, (3) covered the biomechanics, surgical technique, or clinical outcomes of an IPD, and (4) studies were in human patients, cadavers, or biomechanical testing. Exclusion criteria included: case reports, technical notes, non-lumbar spine studies, review articles, animal studies, and editorials/commentaries.

### Biomechanics

#### Enlargement of the spinal canal and foramina

In LSS, symptoms of NCs are exacerbated upon extension of the lumbar spine and relieved on flexion [11]. IPD provides indirect decompression by using intervertebral spacers to enlarge the spinal canal and neural foramina without directly removing stenotic tissue [12, 13]. The implanted spacers physically separate adjacent spinous processes and increase the interspinous distance, described as distracting the spinous processes [12]. A cadaveric study using the X-STOP™ IPD between the L3-L4 spinous processes found that the implant significantly increased the spinal canal area by 18%, subarticular diameter by 50%, canal diameter by 10%, and the foraminal area by 25% [14]. The space-expanding effects of IPD implantation are supported by a clinical MRI study that found that 70.6% of patients treated with the X-STOP™ devices demonstrated an increase in the area of the dural sac from 77.8 mm to 93.4 mm after surgery in the standing position [15]. According to the American Society of Pain and Neuroscience (ASPN), IPDs provide comparable clinical performance to decompressive laminectomy, which removes the spinal lamina to create more space in the spinal canal, in LSS symptom management [16].

The durability of IPD effects on enlarging the spinal canal varies between studies. One study found that IPDs increased dural sac area by 37% one week post-operatively, but this effect was not maintained at 3 months or 2 years after surgery [17]. These findings contrast with a separate study on patients treated with the same model of IPD (X-STOP™), where the dural sac area was maintained at 2 years post-operation, with significant increases in four measured postures (standing, sitting, flexion, and extension) [18]. More studies are needed to examine the magnitude and long-term effects of IPDs on spinal canal dimensions.

#### Effect on the segmental range of motion

While IPDs significantly reduce the extension and stress at the inserted level, they have minimal effect on flexion, lateral bending, and axial rotation, therefore maintaining relatively flexible mobility at the level [19]. Finite element modeling (FEM) has been used to simulate and predict the biomechanical musculoskeletal effects of different spinal implants without testing on cadavers or patients [19]. The model is validated by comparing predictions to published experimental data from cadaveric studies [20]. Across FEM studies, results consistently demonstrate that IPDs significantly reduce the segmental range of motion of extension at the inserted site [19]. One study in particular that tested three different IPD models (Coflex-F™, Device for Intervertebral Assisted Motion (DIAM™, and Wallis™) found that the DIAM™ model behaved most similarly to the intact spine, offering the least restriction to flexion, lateral bending, and torsion, whereas the Coflex-F™ model provided the greatest restriction, particularly in flexion and extension. However, the study found that all IPD models provided a greater range of motion that resembled intact lumbar kinematics than pedicle screw fixation [21]. FEM studies show that IPDs cause adjacent segments to increase their ROM to compensate for restriction at the inserted level; however, these changes are substantially smaller than those observed with rigid fixation, which suggests a reduction in the risk of adjacent segment disease (ASD) [19, 22].

#### IPDs and intradiscal pressure

FEM has also been used to examine the effect of IPDs on intradiscal pressure and facet joint loads [23, 24]. Across several IPD models, IPDs have been found to consistently decrease intradiscal pressure in extension by up to 81% in the Coflex-M™ model. In these studies, intradiscal pressure remains similar to intact spinal values in flexion, lateral bending, and axial rotation, indicating that IPDs selectively reduce disc loading during spinal extension, which is the movement that is most strongly associated with LSS symptom exacerbation [24]. These findings are supported by cadaveric data examining the effect of IPDs on specific subcomponents of intradiscal pressure, including nucleus pulposus and posterior annular ligament pressures [25, 26]. One cadaveric study demonstrated a pressure reduction of 63–73% in the nucleus pulposus pressure and 57–63% posterior annular pressure reduction after IPD placement [26].

#### IPDs and load redistribution

In addition to disc unloading, FEM and cadaveric studies also demonstrate significant reductions in facet joint forces during extension at the level of insertion. A cadaveric study using pressure-sensitive film in the facet joints of the implanted and adjacent levels analyzed after loading, found that IPD implants significantly reduced the mean peak pressure, average pressure, contact area, and force at the implanted level [27]. While IPDs redistribute load away from posterior articulations, it can cause increased forces in the adjacent segments, as modeled in FEM models [28]. In one FEM study, facet loads at adjacent segments increased up to 60% compared to an intact spine, highlighting that while IPD distract force at the spinous processes at the inserted segment, it may affect biomechanical parameters at adjacent levels during extension [22].

However, it is important to note that different IPD models have unique modes of force transfer, which are determined by their structural configuration and material stiffness [28]. Cadaveric studies reveal that the compression stiffness of different models can range from 133 N/mm to up to 1674 N/mm, which also contributes to the variety in force transfer characteristics between models [29].

Notably, to date, there have not been measurements in humans after IPDs for changes in anterior column loads, intradiscal pressure, or pressure gradients in nerves. These measurements are essential in evaluating the mechanistic claim of IPDs.

#### Clinical relevance of biomechanical studies

Biomechanical studies provide insight into the mechanisms through which IPDs can alter spinal kinematics. However, the translation of these findings into a positive clinical benefit is not always certain. FEM and cadaver studies demonstrate a reduction in segmental range of motion at the inserted site, intradiscal pressure, and facet joint forces during extension [19–28]. Theoretically, these changes in spinal biomechanics can contribute to symptom relief in patients with NC.

However, these findings in biomechanical studies may not predict which patients will result in meaningful clinical improvements. For example, it does not take into account the multivariable etiologies to LSS or pain generators, such as from inflammatory mediators or neuropathic and central sensitization [2]. Additionally, biomechanical studies are typically performed with single-level constructs (one motion segment) in a controlled laboratory setting, which does not replicate the physiologic complex dynamic loading, degenerative pathology, or patient-specific anatomy [11].

FEM studies are useful for generating hypotheses, but these rely on an ideal model for material, anatomy, and device positioning that does not reflect real-world variabilities [19, 20]. Cadaver studies, on the other hand, are limited by tissue quality, lack muscle stabilization effects, and are unable to predict changes in pain [28]. Thus, while biomechanical studies are highly reproducible, these limitations preclude physiological accuracy and can lead to issues translating these findings into clinical benefit. Therefore, these studies should be primarily interpreted mechanistically rather than predictively, and robust clinical data are essential in determining if the modeled biomechanical advantages translate into meaningful patient benefit.

### Clinical outcomes

Given the variations in IPDs, the following devices are stratified by design, indication, and surgical approach. Notably, there are limited comparative or head-to-head trials currently existing among these devices, and results should be contextualized to avoid overgeneralization. Table 1 provides a general overview and three categorizations of IPDs: non-fusion, fusion, and hybrid. Since IPDs vary substantially in design, biomechanical intent, surgical approach, indications, and study methodologies, results from one device or device class should not be extrapolated to all IPDs. A summary of clinical outcomes stratified by device can be found in Table 2.

**Table 1:** Interspinous Process Devices Stratified by Design, Indication, and Surgical Approach

Device	Design	Primary Indication	Surgical Approach
BacFuse™	Fusion/Stand-alone	Mild to moderate LSS Degenerative disc disease Grade 1–2 spondylolisthesis	Percutaneous or mini-open lateral or posterolateral approach
Minuteman™			
Spire™			
ZIP™			
Coflex™	Hybrid/Dynamic Stabilization	Moderate to severe LSS Degenerative disc disease Grade 1 spondylolisthesis	Mini-open posterior approach
Wallis™			
DIAM™			
X Stop™	Non-fusion/Motion-preserving	Mild to moderate LSS Grade 1 spondylolisthesis	Percutaneous posterior midline approach
Superion™			
Falena™			
Aperius™			
BacJac™			
Rocker™			
HeliFix™			

**Table 2:** Summary of Clinical Outcomes Stratified by Device ZCQ: Zurich Claudication Questionnaire; SF-36: Medical Outcomes Study Short Form-36; VAS: Visual Analogue Scale; ODI: Oswestry Disability Index; COMI: Core Outcomes Measures Index; JOA: Japanese Orthopaedics Association; PGIC: Patient Global Impression of Change; PROMIS: Patient-Reported Outcomes Measurement Information System; RM: Roland-Morris Disability Questionnaire

Device	Sample Size (*n*)	Mean Follow-up (Months)	Improvement Metrics Reported	Reoperation Rate
BacFuse™	41 to 142	60	ZCQ ↑, VAS ↓, ODI ↓	2.8% to 12.2%
Minuteman™	20	24	ZCQ ↑, VAS ↓, ODI ↓	0%
Spire™	40	14.2	VAS ↓, ODI ↓	5%
ZIP™	69	6	ZCQ ↑, VAS ↓, ODI ↓, PGIC ↑, PROMIS ↑	0%
Coflex™	18 to 42	12 to 60	VAS ↓, ODI ↓	0% to 4.8%
Wallis™	10 to 40	24 to 56	VAS ↓, ODI ↓, RM ↓, SF-36 ↑, JOA ↑	0% to 10%
DIAM™	18	56	VAS ↓, ODI ↓	0%
X Stop™	30 to 201	6 to 24	ZCQ ↑, VAS ↓, ODI ↓, SF-36 ≃	3.3% to 18.9%
Superion™	80 to 190	6 to 24	ZCQ ↑, VAS ↓, ODI ↓	8.8% to 24.2%
Falena™	26 to 35	6 to 12	VAS ↓, ODI ↓	0%
Aperius™	11 to 152	6 to 24	ZCQ ≃, VAS ↓, ODI ↓, COMI ≃, SF-36 ↑	0% to 41.6%
BacJac™	41	36	ODI ↓	14.6%
Rocker™	32	24	ODI ↓, JOA ↑	6.3%
HeliFix™	100	12	VAS ↓, RM ↓	2%

### Non-fusion/motion-preserving IPDs

Non-fusion or motion-preserving IPDs are designed to provide an “indirect decompression” by distracting the spinous process. This mechanism functions to decrease extension with relative preservation of flexion, axial rotation, and lateral bending. The primary indication for this class is for symptomatic improvement of mild to moderate LSS and Grade 1 spondylolisthesis. These devices are typically placed by a percutaneous posterior midline approach. Devices in this category include the X-STOP™, Superion™, Falena™, Aperius™, BacJac™, Rocker™, and HeliFix™.

#### X-STOP™

The X-STOP™ device is made of titanium and was first approved in 2005 for LSS [30]. Zucherman et al. conducted a randomized controlled trial (RCT) comparing the X-STOP™ (100 patients) to non-operative therapy (91 patients). Around 75% of patients at 6 weeks and 6 months had improvement of their symptoms and physical function, and 70% of the patients were satisfied with their treatment. At 1 year, 59% of X-STOP™ patients and 12% of the non-operative patients were significantly improved in the symptom severity and physical function portion of the Zurich Claudication Questionnaire (ZCQ) (*p* < 0.05) [31]. Another study compared X-STOP™ (86 patients) to Superion™ (80 patients). At 6-month follow-up, ZCQ symptom severity scores were 30% improved with Superion™ and 25% with X-STOP™, both with *p* < 0.001. Physical function, measured on the ZCQ, was improved 32% with Superion™ and 27% with X-STOP™, both with *p* < 0.001 [32]. Another study evaluated the long-term outcomes of X-STOP™ up to 4 years after the procedure. Mean Visual analogue scale (VAS) leg pain decreased about 61% preoperatively to about 39% at 6 weeks and was stable until 24 months. ODI decreased from 33% preoperatively to 20% at 24 months. Out of the 175 patients in the study, 8 patients required removal and decompression due to persistent symptoms [33].

#### Superion™

The Superion™ interspinous spacer is made of central titanium with wings that extend cranially and caudally to prevent lateral displacement. The central segment sits between spinous processes [30]. t One study compared Superion™ (190 patients) to X-STOP™ (201 patients) and measured success by using the primary composite endpoint, which was patient success based on four components: improvement in two of the three ZCQ domains, no reoperations, no major procedural complications, and no clinically significant confounding treatments. At 2 years, 52.5% of patients who received Superion™ and 38% of patients who received X-STOP™ achieved the primary composite endpoint (*p* = 0.023) [34]. Leg pain was decreased by 70%, and 77% of patients achieved leg pain clinical success (improvement ≥ 20 mm). Back pain clinical success (improvement ≥ 20 mm) was achieved in 68%, and ODI improved in 65% of patients [34]. At 4 years, 84.3% of patients showed improvement in greater than or equal to 2 of the 3 ZCQ domains, 62% had improvements in ODI, and 66% improvement in VAS back and leg pain 75% of patients were free from reoperation, revision, or additional fixation at 4 years [35].

#### Falena™

The Falena™ IPD is made from polyetheretherketone (PEEK), similar to the ZIP™ device [36]. One study compared the Falena™ IPD (35 patients) to the Aperius™ IPD (24 patients). Both groups showed a statistically significant decrease in the VAS and ODI scores at 6 months and 12 months, with *p* < 0.0001. There was also a statistically significant increase in vertebral canal area (Falena™ *p* < 0.0001 and Aperius™ *p* = 0.0003). The main difference between these two groups was found at the one-year follow-up, where there was a higher increase of vertebral canal area in patients who received the Falena™ device (*p* < 0.001) [37].

#### Aperius™

Aperius™ is made out of a titanium alloy core with a pure titanium shell. It was designed to be a standalone implant that does not require the removal of the interspinous ligament [30]. Beyer et al. compared the Aperius™ device (12 patients) to bilateral open microsurgical decompression (33 patients). At 2 years, the patients who underwent open decompression reported significant improvements in back and leg pain and ODI, which was not observed in the Aperius™ group. However, walking distance improved in both groups [38]. At two years, five patients who had the Aperius™ device implanted required implant removal and subsequent open decompression. Another study compared the Aperius™ spacer to microsurgical bilateral decompression. There was no statistical significance in the ODI in the Aperius™ group at any point in follow-up, but VAS leg pain was significantly improved at 6 months (*p* < 0.05); however, this was no longer the case at one year. Walking time was significantly improved in both groups at one-year follow-up. There was a 27.3% reoperation rate for the Aperius™ group and 0% reoperation rate for the decompression group [39]. On another note, one study looked at the effectiveness of the Aperius™ device in 70 patients for 6 months. VAS pain scores improved on average from 8.2 to 3.6, the overall patient satisfaction was 76%, and no complications were noted [40]. Another study looked at the effectiveness of Aperius™ for NC in 152 patients with no adverse events noted. There was also a significant improvement in VAS and ZCQ for low back and leg pain and for NC [41].

#### BacJac™

The BacJac™ device is made out of PEEK and aims to preserve the supraspinous ligament [12]. Spallone et al. studied the effectiveness of the BacJac™ device in 41 patients. All patients showed significant improvement in the ODI score immediately after surgery; however, only 41% of patients reported a satisfactory outcome. They found worse results when patients were operated on at the L3-4 level [42].

#### Rocker™

The Rocker™ IPD is manufactured from titanium and can be installed using a unilateral approach. One study compared the Rocker™ device (32 patients) to X-STOP™ (30 patients). Both groups showed significant improvement in ODI scores at 24 months. Two patients treated with the Rocker™ device had implant dislocation within one week, and one patient in the X-STOP™ group had implant migration in two months [43].

#### HeliFix™

The HeliFix™ device is inserted into the interspinous space after opening the interspinous ligament. The device has a self-distracting helical tip that is inserted into the interspinous space and works with the supraspinous ligament to keep the device in place. Alexandre et al. examined the effectiveness of the HeliFix™ device over 12 months in 100 patients. They found leg and back pain, functional improvement, and decreased usage of pain medication. In 2% of patients, the device was removed due to persistent pain [44].

### Fusion/stand-alone IPDs

Stand-alone fusion IPDs function mechanistically differently from non-fusion IPDs, as these are fundamentally rigid implants that anchor onto the spinous processes. Unlike non-fusion IPDs, the goal of fusion IPDs is to achieve arthrodesis between adjacent spinous processes and restrict motion in flexion, extension, lateral bending, and axial rotation. The primary indication for this class is for mild to moderate LSS, degenerative disc disease, and Grade 1 to 2 spondylolisthesis. These devices are typically implanted using a percutaneous or mini-open lateral or posterolateral approach. The fixation is performed using an interspinous bone graft to facilitate fusion. Devices in this category include the BacFuse™, Minuteman™, Spire™, and ZIP™.

#### BacFuse™

BacFuse™ is an IPD that can be used to distract the spinous process and achieve fusion of the posterior column. A bone graft can be used to promote the interspinous process fusion [45]. BacFuse™ showed statistically significant reduction in VAS and ODI scores in the immediate postoperative period ( *p* < 0.05) and sustained in 32 of 41 patients at 2-year follow-up. In the short term, symptom improvement was most pronounced in patients with moderate LSS [46]. A survey found that 24 patients were very satisfied, and 9 were not satisfied with the results of the surgery [46]. Another study looked at 5-year follow-up outcomes by stenosis type (stand-alone fusion and a decompression-combined group). There was no significant difference between the groups in the moderate lateral stenosis subtype, with 93.33% satisfaction in the stand-alone group and 95.65% in the decompression combined group. Patients with severe lateral recess stenosis showed a 90.63% satisfaction rate in the decompression combined group and only 65.22% in the stand-alone group. Those with severe central stenosis showed 57.14% satisfaction in the stand-alone group and 54.55% in the decompression group [45].

#### Minuteman™

Minuteman™ consists of a central body with two wings near the distal end and a cap on the proximal end. The body of the device provides indirect decompression and carries bone graft fusion material. In a comparative study between Minuteman™ and standard surgical decompression, Minuteman™ showed significant improvement in leg pain, back pain, ODI, and ZCQ compared to baseline at follow-up from 8 weeks to 24 months. Many patients with the IPD device showed early functional recovery and were walking 30 min post-procedure. There was a statistically significant difference between the decompression alone group at the 24-month follow-up for leg pain, ODI, and physical function (*p* < 0.001). At 24 months, improvement of back pain was equal between the groups [47].

#### Spire™

The Spire™ is made of titanium alloy [48]. One study compared the Spire™ (40 patients) to a pedicle screw fixation group (36 patients). There was a statistically significant reduction in VAS pain scores and ODI in both groups at one year after surgery, suggesting comparable clinical efficacy of the Spire™ to pedicle screw fixation (*p* < 0.05). ASD occurred more in the pedicle screw group, and this difference was statistically significant, which suggests reduced biomechanical stress on adjacent levels (*p* = 0.029) [49].

#### ZIP™

The ZIP™ IPD is made from PEEK, which provides structural support while maintaining some compliance [20]. One study analyzed the ZIP™ device at a 90-day and 6-month follow-up. There was a 62% improvement in VAS back pain and a 64% improvement in VAS leg pain. The ZCQ improvement was 76%, and the ODI improvement was 64%. The Patient Global Impression Change (PGIC) was used to assess patient-reported improvement, and 78% of patients reported improvement. There was at least a 5-point improvement in all seven domains in the Patient-Reported Outcomes Measurement Information System (PROMIS). Improvements were stable when assessed at 90 days and 6 months [50].

### Hybrid/dynamic stabilization IPDs

Hybrid or dynamic stabilization IPDs offer a middle ground between non-fusion and fusion IPDs and are typically implanted following surgical decompression. These devices are designed to reduce extension but provide “dynamic stabilization” through limited reduction in flexion, lateral bending, and axial rotation. The primary indications for this class are moderate to severe LSS, degenerative disc disease, and Grade 1 spondylolisthesis. These devices are typically implanted using a mini-open posterior approach. This category includes Coflex™, Wallis™, and DIAM™.

#### Coflex™

The Coflex™ device is U-shaped and made of titanium. It is placed between adjacent laminae and spinous processes after decompression surgery. It preserves both flexion and extension in patients with LSS [30]. One study compared implanting a Coflex™ device (18 patients) to posterior lumbar interbody fusion (PLIF) (24 patients). At a 12-month follow-up, both groups showed significant improvement in VAS and ODI scores for back and leg pain (*p* < 0.05), but no difference was observed between groups [51]. Yuan et al. found that patients with the Coflex™ device (42 patients) had less blood loss in surgery and shorter hospital stays compared to PLIF (45 patients). Both groups showed significant improvement in VAS and ODI scores for back and leg pain at each follow-up, for up to 5 years (*p* < 0.001). There was a lower reoperation rate for ASD in the Coflex™ group (4.8%) than the PLIF group (11.1%); however, this difference was not statistically significant (*p* = 0.277) [52].

#### Wallis™

The Wallis™ device was originally made from titanium and can be used for restoration or preservation of lumbar motion. It may also be used as an adjunct to decompressive surgery. The second-generation device consists of two Dacron ribbons to limit flexion and a PEEK spacer to limit extension. The implant is inserted between spinous processes, and the ribbons are wrapped around the adjacent spinous processes [30]. One study evaluated the Wallis™ device at a 24-month follow-up; there was a statistically significant decrease in VAS back pain (*p* = 0.017) and VAS leg pain (*p* = 0.006). ODI also decreased from 40.0% preoperatively to 17.3% [53]. Korovessis et al. compared the Wallis™ (17 patients) to the DIAM™ (18 patients). The DIAM™ device is made out of silicon [14]. Both groups had statistically significant improvement in clinical outcomes at six months (*p* = 0.001). No difference was found in VAS or ODI scores between the two devices [54]. Another study looked at the effects of lumbar discectomy plus the Wallis™ (40 patients) versus lumbar discectomy alone (37 patients). At the three-year follow-up, there was no significant difference between the groups in VAS or ODI, with *p* > 0.05. The Wallis™ group had significantly greater disc height (*p* < 0.001). There was a similar reoperation rate between groups, two in the Wallis™ group and three in the control group [55].

### Safety

Compared with traditional laminectomy, IPDs offer shorter procedure times, the ability to be performed under local anesthesia, minimal blood loss, and reduced risks of CSF leak [56]. However, these advantages do not necessarily translate into superior or durable clinical outcomes, as systematic reviews have demonstrated higher reoperation rates for IPDs compared with conventional decompression, despite similar short-term pain relief [10, 57].

The Felix trial was one of the first large RCTs to compare the clinical outcomes and safety of standalone IPD and traditional bony decompression [58]. Results from the study noted that there was no significant difference in VAS back and leg pain, with higher reoperation rates with IPD compared to decompression (33% versus 8%, respectively). Furthermore, a 2024 Manufacturer and User Facility Device Experience (MAUDE) database analysis assessed the adverse events associated with IPDs. Of the 943 surgery-related adverse events, 374 were attributed to device malfunction and 251 to persistent postoperative pain [59]. Importantly, 48.8% of reported adverse events ultimately led to revision surgery.

Notably, the X-STOP™ was withdrawn from the U.S. market in 2015 due to its unfavorable safety profile [60]. In an FDA MAUDE database analysis (2010–2020), X-STOP™ accounted for 245 adverse event reports, including spinous process fractures, device migration or dislodgement, implant loosening or breakage, or recurrent back and leg pain [56]. In a separate long-term study of 12 patients with lumbar stenosis and mild spondylolisthesis treated with X-STOP™, 8 patients initially reported complete symptom relief. However, within 2 years, nearly 25% reported recurrence of symptoms and 58% required salvage decompression and fusion [61].

The Superion™ is a second-generation IPD designed for percutaneous placement, receiving FDA approval in 2015. Despite design modifications, the Superion™, like its predecessor, has several reported complications, including spinous process fractures and device malposition. In the 2024 MAUDE analysis, implant malfunctions accounted for nearly half of the adverse events associated with Superion™, highlighting persistent safety challenges [59].

In a 2017 review, Pintauro et al. found a mean reoperation rate of 13.35% at a mean follow-up of 24 months. However, the next-generation IPDs had a lower reoperation rate of 3.7% compared to 11.1% for the first-generation IPDs [35]. The most common causes of reoperations were spinous process fractures, device migration/loosening, radicular deficits, and recurrent/persistent symptoms.

By altering the biomechanics at the instrumented level, IPDs may also impact neighboring segments. Rigid spacers may increase stress on the intervertebral discs and facet joints at adjacent levels. Increased adjacent-level stress may contribute to accelerated degeneration, instability, and spinous process fracture [19]. As a result, increased stress in adjacent levels may contribute to ASD, instability, and spinous process fracture. To reduce the risk of reoperations, patients with osteopenia/osteoporosis, ASD, severe canal/foraminal stenosis, or isthmic spondylolisthesis should not have an IPD placed [35].

### Industry involvement

Because many interspinous devices were developed and marketed by device companies, potential industry bias in the research has been a significant concern (Table 3). The literature has been closely examined for conflicts of interest and their impact on reported outcomes and study interpretations. Several pivotal trials for IPDs that led to regulatory approvals for devices such as X-STOP™ and Superion™ were industry-sponsored or had authors who had consulting relationships [31, 34]. It’s important to note that these influences do not inherently invalidate the results but rather highlight the importance of interpreting the reported outcomes in light of their conflicts of interest.

**Table 3:** Interspinous Spacer and Fusion Trials with Disclosure of Sponsorship and Conflicts of Interest

Study	Device	Funding Source	Disclosures
*Zucherman*,* 2005 *[62]	Interspinous Spacer (X-Stop™)	Industry (unnamed)	One or more authors has/have received or will receive benefits from a commercial party related to the manuscript topic.
*Kong*,* 2007 *[51]	Interspinous Spacer (Coflex™)	Research Grant (IN-SUNG Foundation for Medical Research)	No conflict of interest reported.
*Kuchta*,* 2009 *[33]	Interspinous Spacer (X-Stop™)	No funding noted	No conflict of interest reported.
*Menchetti*,* 2010 *[40]	Interspinous Spacer (Aperius™)	No funding noted	No conflict of interest reported.
*Nardi*,* 2010 *[41]	Interspinous Spacer (Aperius™)	No funding noted	No conflict of interest reported.
*Sobottke*,* 2010 *[39]	Interspinous Spacer (Aperius™)	No funding noted	One author was a consultant to Medtronic
*Shabat*,* 2011 *[63]	Interspinous Spacer (Superion™)	Industry (Vertiflex, Inc)	No conflict of interest reported.
*Miller*,* 2012 *[32]	Interspinous Spacer (X-Stop™)	No funding noted	No conflict of interest reported.
*Beyer*,* 2013 *[38]	Interspinous Spacer (Aperius™)	Government research grant (German Federal Ministry of Research and Education)	No conflict of interest reported.
*Davis*,* 2013 *[64]	Interspinous Spacer (Coflex™)	Industry (Paradigm Spine, LLC)	Potential COI with additional financial activities regarding payment for consulting, lectures, manuscript preparation, educational presentations, and royalties.
*Alexandre 2014 *[44]	Interspinous Spacer (Helix™)	No funding noted	No conflict of interest reported.
*Patel*,* 2014 *[65]	Interspinous Spacer (Superion™, X-Stop™)	Industry (Vertiflex, Inc)	Two authors are consultants to VertiFlex, Inc.
*Puzzilli*,* 2014 *[66]	Interspinous Spacer (X-Stop™)	Independent	No conflict of interest reported.
*Huang*,* 2015 *[43]	Interspinous Spacer (Rocker™)	No funding noted	No conflict of Interest reported.
*Lønne*,* 2015 *[67]	Interspinous Spacer (X-Stop™)	Independent	No conflict of interest reported.
*Patel*,* 2015 *[34]	Interspinous Spacer (Superion™, X-Stop™)	Unnamed sponsor	Potential COI as data were regularly monitored by the sponsor.
*Daentzer*,* 2016 *[53]	Interspinous Spacer (Wallis™)	Industry (Zimmer Spine SAS)	No conflict of interest reported.
*Ghany*,* 2016 *[68]	Interspinous Spacer (Coflex™, X-Stop™)	Independent	No conflict of interest reported.
*Masala*,* 2016 *[37]	Interspinous Spacer (Falena™)	Independent	No conflict of interest reported.
*Meyer*, 2017 [69]	Interspinous Spacer (Aperius™)	Industry (Medtronic Spinal and Biologics)	One author has been a consultant for Medtronic outside of the study.
*Nunley*,* 2017 *[35]	Interspinous Spacer (Superion™)	Industry (Vertiflex, Inc)	No conflict of interest reported.
*Strömqvist*,* 2017 *[70]	Interspinous Spacer (X-Stop™)	Independent	No conflict of interest reported.
*Yuan*,* 2017 *[52]	Interspinous Spacer (Coflex™)	Independent	No conflict of interest reported.
*Gu*,* 2018 *[55]	Interspinous Spacer (Wallis™)	Government research grant (Science and Technology Planning Project of Guangdong Province, China)	No conflict of interest reported.
*Korovessis*,* 2018 *[54]	Interspinous Spacer (Wallis™)	No funding noted	No conflict of interest reported.
*Spallone*,* 2019 *[42]	Interspinous Spacer (BacJac™)	No funding noted	No conflict of interest reported.
*Jung Kim*,* 2012 *[71]	Interspinous Fusion Device (Spire™)	Independent	No conflict of interest reported.
*Chen*,* 2020 *[45]	Interspinous Fusion Device (BacFuse™)	Independent	No conflict of interest reported.
*Spallone*,* 2022 *[46]	Interspinous Fusion Device (BacFuse™)	Government research grant (Ministry of Higher Education of the Russian Federation)	No conflict of interest reported.
*Falowski*,* 2023 *[50]	Interspinous Fusion Device (ZIP™)	Industry (multiple medical device companies)	Several authors report financial relationships with numerous medical device and pharmaceutical companies, including consulting roles, speaker honoraria, research grants, and educational support. Some hold stock, equity, or royalties in various firms. Several companies, including Aurora Spine (maker of ZIP) are mentioned in these disclosures, with one author also reporting a licensed patent.
*Baranidharan*,* 2024 *[47]	Interspinous Fusion Device (Minuteman™)	Industry (Spinal Simplicity)	One author is part of the medical advisory board with stock options for Spinal Simplicity. One author reports personal fees from Spinal Simplicity, outside the submitted work, and two authors report personal fees during the study.

Broader orthopedic literature also reflects this concern. In a review of 100 RCTs published in leading orthopedic journals, one study identified a strong association between industry sponsorship and the likelihood of positive trial outcomes. Approximately 85% of the reviewed industry-funded trials reported favorable results for the investigated treatment, a proportion significantly higher than non-industry trials (*p* < 0.001) [72]. Additional spine-specific research has supported these findings, noting that authors with financial disclosures are less likely to publish studies with neutral or negative conclusions [73]. Systematic reviews have similarly reported that a substantial portion of IPD studies are industry-sponsored. While industry partnerships remain important to continue the advancement of medical technology, there is a continued need for objective studies and independently funded research [74]. Collectively, these findings suggest that industry ties may influence the tone and interpretation of study results, reinforcing the importance of critical appraisal and careful consideration of author disclosures when evaluating the IPD literature.

### Clinical implications

Pain and symptoms from LSS are often multifactorial, including factors like mechanical compression, nociceptive back pain, NC, and neuropathic or centrally mediated pain [75]. Hence, structural correction alone might not translate to effective long-term symptom relief, particularly for patients with complex or chronic pain presentations. The discrepancy between clinical outcomes and anatomic changes suggests a significant consideration: Even though IPDs could improve some posture-dependent symptoms through reducing extension-induced neural compression, chronic or centrally mediated pain mechanisms could continue to persist in patients despite biomechanical decompression [75]. Thus, although NC symptoms may improve initially, it does not treat chronic radicular pain.

Population studies and analysis highlight the complexity of IPDs. In a national claims database study consisting of 4,865 patients who underwent IPD implantation, the 3-year reoperation rate was approximately 10%, with higher reoperation rates observed in patients undergoing concomitant open decompression [76]. Other reviews have suggested similar concerns of increased reintervention rates and evaluated cost-effectiveness when compared to traditional decompression procedures [57]. With regards to cost, IPD can be more expensive or have no cost-effective differences compared to decompression surgery [35].

Due to the complexity of symptoms of patients with LSS, patient selection is critical. Current literature consistently encourages IPD use in patients who have posture-dependent and moderate NC affecting 1–2 levels without major spinal instability, chronic radiculopathy, or spondylolisthesis [10]. On the other hand, patients who have chronic radicular symptoms or central pain might benefit less from IPDs, due to how the pain mechanism extends beyond mechanical compression alone. Clinically, current evidence shows that IPDs could serve as an alternative option within a broad treatment regimen in selected patient populations. Combining assessment of pain pattern, functional impairment, central pain contributions, and radiographic results could provide more tailored decision-making for patients. Clinicians should discuss short-term symptom relief of IPDs, limitations of indirect decompression, and possible subsequent surgical intervention, along with an evidence-informed approach to LSS management.

### Future directions and recommendations

Current evidence on IPDs is limited by short follow-up periods and biases resulting from study design [10]. Thus, there is a clear need for independent, well-powered RCTs with long-term follow-up beyond 5 years. A longer follow-up period is necessary as early benefits have been shown to diminish over time. For instance, one long-term IPD study reported that, by final follow-up (7 years), 80% of patients had average or poor outcomes and two-thirds required reoperation [77]. Future studies should follow outcomes at 5–10 years or more to better assess the sustainability of symptom relief and reoperation rates.

Furthermore, recent systematic reviews suggest that future trials should closely follow CONSORT guidelines and incorporate more rigorous methodologies to minimize bias [10]. Many initial IPD trials were found to have a high risk of performance bias, attrition bias, and incomplete data reporting, making it difficult to draw firm conclusions on the effectiveness of these devices [10]. To reduce the risk of bias, future studies should include proper randomization/blinding, independent funding, follow standardized protocols, and transparently report all outcomes, even if they are unfavorable.

Most studies to date have focused on patient-reported outcomes (PROs), including pain scores (VAS), the ODI, and ZCQ [45]. While PROs are valuable measures, future studies should include objective functional outcomes to complement this data. This includes physical performance measures such as walking distance, return to activity/sport, and biomechanical studies on load distribution or disc pressures. Such outcomes can better suggest how well IPD treatment helps patients in their day-to-day lives over time.

### Limitations

This data must be contextualized with the limitations of this study. First, the study design is a narrative review, not a systematic review, without pooling outcomes or meta-analytic methodology. This was due to the current literature on IPDs having significant heterogeneity with design, biomechanics, operative technique, outcomes reported, and follow-up duration. These studies also had small cohort sizes, short follow-up periods, or lacked independent funding, which may introduce bias. Thus, the long-term data remains unclear, limiting the ability to pool outcome data by devices or provide definitive conclusions based on the available data. These limitations should be considered when interpreting the findings summarized and presented in this review.

## Conclusion

Standalone IPDs represent a diverse variety of implants with distinct biomechanical mechanisms, techniques, and clinical indications. Current evidence suggests that some devices may provide short-term symptomatic improvement in carefully selected patients. Due to the heterogeneity between devices, outcomes from one device type should not be generalized to others within the class. However, long-term benefits in pain, function, or quality of life are unclear, and several IPDs have demonstrated higher reoperation rates compared to standard surgical decompression. Mechanical “indirect” decompression of the neuroforamen does not treat chronic radicular pain, as these mechanisms may be neuropathic and/or centrally sensitized. Future studies may better define an appropriate patient population for IPDs, such as those who cannot undergo surgical decompression. However, without independent and robust long-term trials, standalone IPDs may be de-emphasized compared to established surgical treatments.

## Data Availability

No datasets were generated or analysed during the current study.

## Abbreviations

LSS: lumbar spinal stenosis
AP: anteroposterior
CT: computed tomography
NSAIDS: nonsteroidal anti-inflammatory drugs
MIST: minimally invasive spine treatment
ODI: Oswestry Disability Index
IPDs: interspinous process devices
FDA: Food and Drug Administration
NC: neurogenic claudication
MRI: magnetic resonance imaging
ASPN: American Society of Pain and Neuroscience
FEM: Finite element modeling
RCT: randomized controlled trials
ASD: adjacent segment degeneration
VAS: visual analogue scale
ZCQ: Zurich Claudication Questionnaire
PEEK: polyeheretherketone
PGIC: Patient Global Impression Change
PROMIS: Patient-Reported Outcomes Measurement Information System
PLIF: posterior lumbar interbody fusion
MAUDE: Manufacturer and User Facility Device Experience
PROs: patient-reported outcomes
